# The role of sea buckthorn in skin and mucosal health: a review from an anti-inflammatory perspective

**DOI:** 10.3389/fphar.2025.1643146

**Published:** 2025-11-11

**Authors:** Xiayinan Song, Xinyao Sun, Huimin Yuan, Yang Tang, Fengjie Zheng

**Affiliations:** School of Traditional Chinese Medicine, Beijing University of Chinese Medicine, Beijing, China

**Keywords:** sea buckthorn, inflammation, skin, mucosal, nutraceuticals

## Abstract

The skin and mucosal barriers serve as essential frontline defenses, protecting against pathogens, environmental insults, and excessive water loss while maintaining physiological homeostasis. Sea buckthorn (*Hippophae rhamnoides*), a plant long utilized in traditional medicine, has recently garnered scientific attention for its therapeutic potential in enhancing barrier integrity. Modern studies reveal that its bioactive compounds—including flavonoids, unsaturated fatty acids, vitamins, and carotenoids—exert multifaceted pharmacological effects, such as anti-inflammatory, antioxidant, and tissue-repair properties. These mechanisms not only reinforce barrier function but also mitigate inflammation and accelerate healing. This review synthesizes current evidence on sea buckthorn’s multi-target anti-inflammatory actions and its implications for skin and mucosal health through a unique lens of the inflammatory cascade. By elucidating its molecular and cellular effects across distinct stages of inflammation, we provide a foundation for translating these insights into novel dermatological and mucosal therapeutics. The findings underscore the untapped potential of natural products in barrier protection and regenerative medicine, paving the way for future clinical applications.

## 1 Introduction

The skin and mucosal barriers serve as the body’s frontline defense, employing integrated physical, chemical, and microbiological mechanisms to maintain homeostasis. Recent advances highlight the skin’s structural role as a protective shield (Saheb Kashaf et al., 2022), while mucosal surfaces are now recognized as dynamic immunoregulatory interfaces with systemic physiological influence (Graham et al., 2019; Gnirck et al., 2023; Weight et al., 2019).

Research, both domestic and international, has linked dysfunctions in these barriers to numerous diseases, including atopic dermatitis (AD), psoriasis (PSO), ichthyosis, ulcerative colitis, and chronic sinusitis. These chronic inflammatory disorders share common hallmarks: persistent immune activation, recurrent flares, and frequent comorbidities. Despite their clinical prevalence, the molecular mechanisms remain incompletely characterized. Current therapies often focus on symptom suppression rather than mechanistic resolution, resulting in treatment dependence and suboptimal outcomes. Moreover, psychosocial and lifestyle determinants are frequently neglected in clinical management. This underscores the urgent need for novel therapeutics that combine mechanistic precision, favorable safety profiles, and patient-centric approaches. Natural product-derived compounds offer particular promise in this context.

China’s rich tradition of **metabolite** and biodiversity provides a robust platform for natural product development. Notable examples include sea buckthorn, amaranth (Zhao et al., 2015), astragalus (Yang et al., 2020) and scutellaria (Li Y.-Y. et al., 2022) —all demonstrating documented barrier-protective effects. Among these, sea buckthorn (*Hippophae rhamnoides L.*) stands out as a phylogenetically ancient Elaeagnaceae species with a 200-million-year evolutionary history (Janceva et al., 2022). Its medicinal use dates back centuries, with modern applications spanning pharmaceuticals, nutraceuticals, and cosmeceuticals. The fruit’s phytochemical richness—including flavonoids, phenolic metabolites, vitamins, and essential fatty acids (Singh and Sharma, 2022) —underpins its multimodal bioactivity: anti-inflammatory, antioxidant, metabolic regulatory, and organoprotective effects (Sireswar et al., 2020; He Q. et al., 2023; Dupak et al., 2022; Yasukawa et al., 2009; Gęgotek et al., 2018; Luo et al., 2015; Sun et al., 2023; Cheng et al., 2003). Emerging evidence particularly highlights its potential for barrier reinforcement, mediated through antimicrobial, anti-inflammatory, and tissue-repair mechanisms.

**FIGURE 1:**
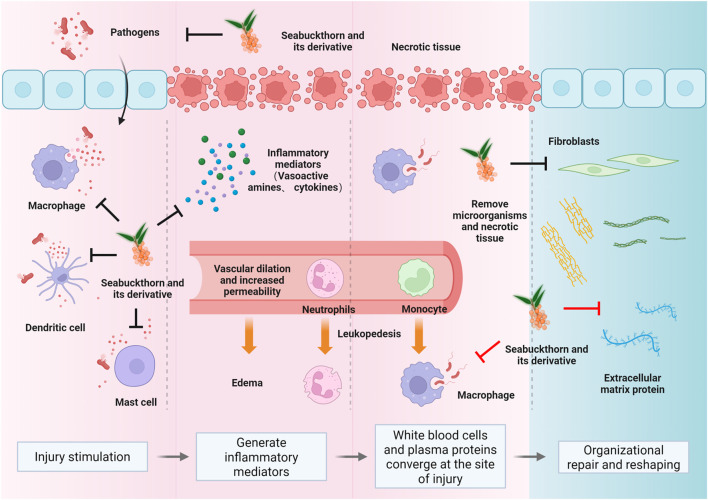
The fundamental pathological changes in inflammation encompass degeneration, exudation, and proliferation (Peña and Martin, 2024; Guo and DiPietro, 2010). These interconnected processes form the dynamic nature of inflammation. Sea buckthorn’s modulatory effects on skin and mucosal inflammation span the entire spectrum of inflammatory injury, anti-injury, and repair (Chen A. et al., 2024; Dilber et al., 2023). Key mechanisms include the elimination of inflammatory stimuli (primarily physical and biological factors) (Smida et al., 2019; Akhtar et al., 2010), blockade of pro-inflammatory signaling pathways (such as NF-κB, MAPK) (Lam and Baumgarth, 2023; Zhao et al., 2020a; Kwon et al., 2012), inhibition of inflammatory mediator production (including Th1, Th17, and the MMP family) (Wang X. et al., 2020; Wu et al., 2022), and the promotion of tissue repair and remodeling once inflammation subsides (Seven et al., 2009).

While existing literature has broadly cataloged the nutritional and antioxidant value of sea buckthorn, this review seeks to deepen the discourse by systematically synthesizing evidence from the specific perspective of the inflammatory cascade. Inflammation involves a coordinated sequence of events, from pathogen recognition and immune activation to the release of mediators and finally, tissue repair and remodeling. This review categorizes sea buckthorn’s anti-inflammatory effects into four interconnected mechanisms that correspond to these key stages: stimulus removal, suppression of pro-inflammatory signaling, pro-inflammatory mediator catabolism, and tissue repair/remodeling (see Figure 1). This framework addresses a critical gap by providing a unified understanding of its therapeutic potential in chronic inflammatory conditions where barrier dysfunction is a hallmark.

This study investigates sea buckthorn’s protective effects on cutaneous and mucosal barriers, aiming to elucidate fundamental inflammatory pathways. Our findings may inform novel therapeutic strategies for chronic inflammatory disorders, bridging traditional knowledge with contemporary mechanistic understanding.

## 2 Overview of sea buckthorn and its derivatives

The taxonomy of sea buckthorn is complex, but according to the authoritative Plants of the World Online database, *Hippophae rhamnoides* L. (Elaeagnaceae) is recognized as a single species with seven accepted infraspecific taxa (all at the rank of subspecies): *H. rhamnoides* subsp. *Carpatica*, subsp. *Caucasica*, subsp. *Mongolica*, subsp. *Rhamnoides*, subsp. *Turkestanica*, subsp. *Wolongensis*, and subsp. *Yunnanensis*. This species is native to a broad trans-Palearctic region, including countries from Central Europe (e.g., Austria, Germany) to East Asia (e.g., China, Mongolia) (Shen et al., 2022; He et al., 2016). Recognized in the *Pharmacopoeia of the People’s Republic of China* (2020 edition), its dried fruits exhibit multifaceted pharmacological activities, including spleen-strengthening, digestive promotion, antitussive/expectorant effects, and blood stasis resolution (Liu et al., 2024). The modern processing of mature sea buckthorn fruits primarily involves mechanical pressing to separate the juice and pomace, followed by CO_2_ supercritical fluid extraction to obtain seed oil and fruit oil from the seeds and peel, respectively; traditional medicinal processing often involves sun-drying and decoction for oral administration, or slow simmering to extract oil, or directly crushing fresh fruits for external application. The plant can be processed into various medicinal preparations, including fresh fruits, extracts, juice, flavonoids, and seed oil/pulp oil. Modern phytochemical analyses indicate that its berries, seeds, leaves, and juice all exhibit significant bioactivity potential (Tiitinen et al., 2005; Arimboor et al., 2008; Fang et al., 2013).

Flavonoids, the predominant phenolic metabolites, serve as quality control markers (Tan et al., 2022), exhibiting tissue-specific distribution: leaves contain the highest concentrations, followed by pulp, whole fruit residue, pericarp, and seeds (Barl et al., 2002; Šne et al., 2013; Kreps et al., 2021). Correspondingly, antioxidant capacity follows this hierarchy, with leaves outperforming stems and fruits (Sytařová et al., 2020).

These findings position sea buckthorn as a promising model for targeted therapeutic development. Its diverse bioactive profile enables precision modulation of specific pathways—akin to “signaling bias” in drug design—to optimize efficacy while minimizing off-target effects.

## 3 Mechanisms of sea buckthorn and its derivatives in the regulation of inflammation in skin and mucous membranes

**FIGURE 2:**
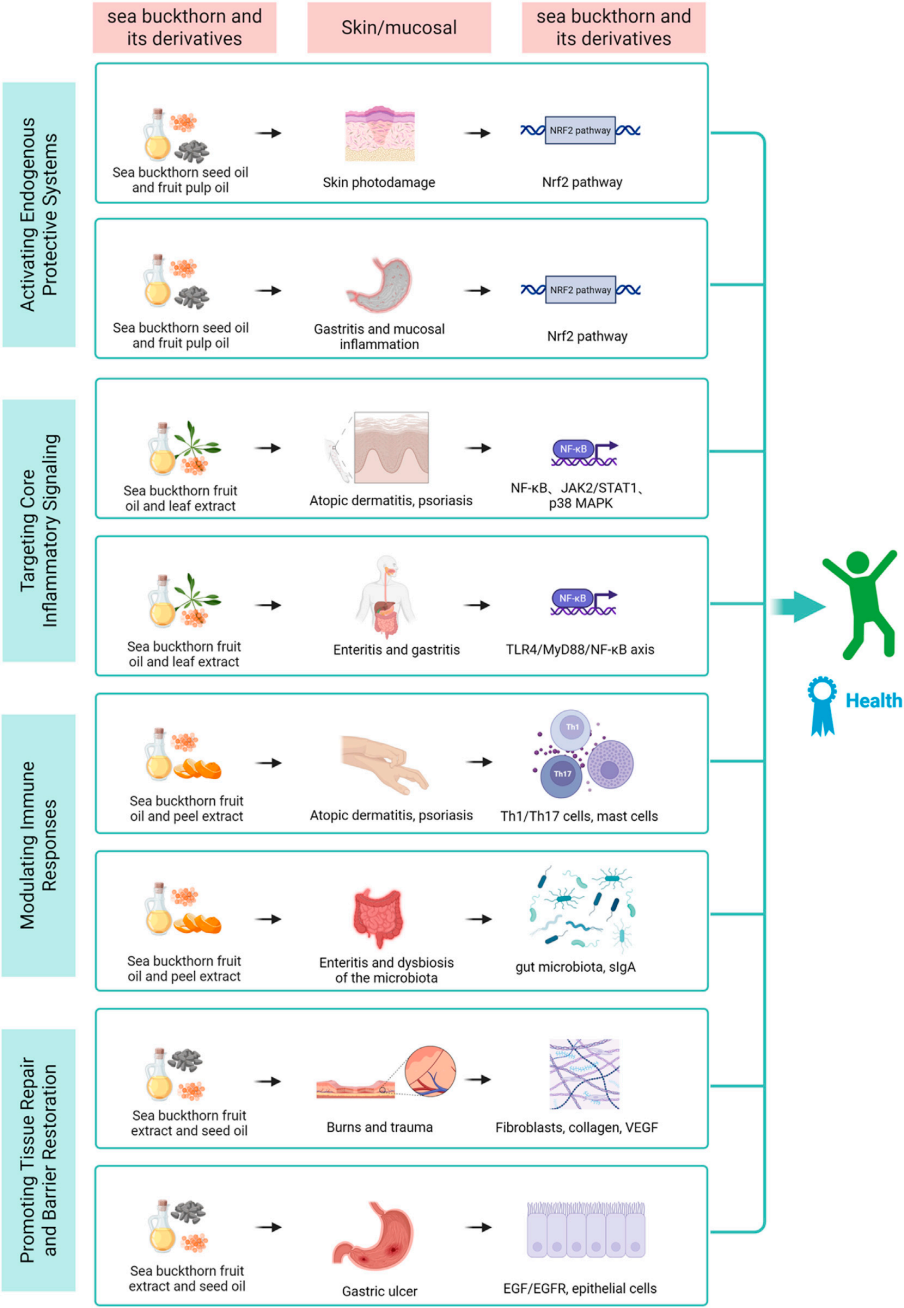
This diagram illustrates the four core mechanisms through which sea buckthorn bioactives synergistically act on molecular pathways and cellular targets in skin and mucosal barriers. By activating antioxidant defense, inhibiting inflammatory signaling, modulating immune balance, and promoting tissue repair, it exerts tissue-specific protective effects, ultimately achieving anti-inflammation, barrier restoration, and tissue homeostasis, revealing its therapeutic potential for related chronic inflammatory diseases.

Sea buckthorn exerts its protective effects on skin and mucosal barriers by intervening at multiple nodes of the inflammatory network. Its bioactives intervene at sequential stages of the inflammatory cascade: activating antioxidant defenses to counter initial stimuli, suppressing core signaling hubs to curb amplification, modulating immune cell responses to balance the reaction, and promoting tissue repair to restore homeostasis (see Figure 2). The following sections detail these mechanisms, highlighting both shared pathways and tissue-specific actions in skin and mucosal (Nathan, 2002).

### 3.1 Activation of protective systems: The Nrf2 antioxidant pathway

Oxidative stress is a common driver and amplifier of inflammation (Visan, 2023). The nuclear red-family 2-related factor (Nrf2) pathway is a central regulator of cellular antioxidant defenses (Hybertson et al., 2011). Activating this endogenous system is a key strategy to mitigate oxidative damage, which is both a cause and consequence of inflammation (Pérez-Torres et al., 2017). Sea buckthorn activates this system to mitigate oxidative damage in both skin and mucosa, though the stressors differ.

#### 3.1.1 Photoprotection and antioxidant defense in skin

Ultraviolet (UV) radiation is a primary oxidative stressor for skin (Rinnerthaler et al., 2015; Poplová et al., 2023). Sea buckthorn seed oil has demonstrated potential as a natural skin photoprotective metabolite, reducing UV irradiation-induced inflammatory responses by protecting the redox balance and lipid metabolism of skin cells, increasing antioxidant levels, and inhibiting the production of lipid peroxidation products. This effect is mediated through the upregulation of Nrf2 activity (Gęgotek et al., 2018). Further supporting this mechanism, a recent study utilizing primary human dermal fibroblasts (HDFs) and Balb/c mouse models revealed that Naringenin—a flavonoid isolated from sea buckthorn fruit pulp—effectively alleviates UV-B-induced oxidative stress and inflammation. Specifically, Naringenin reduced oxidative stress by scavenging reactive oxygen species (ROS) and upregulating key antioxidant enzymes, including catalase and Nrf2. Concurrently, it restored mitochondrial dynamics balance by reducing fragmentation and suppressed the phosphorylation of nuclear factor-kappa B (NF-κB), thereby blocking the production of pro-inflammatory cytokines (Sajeeda et al., 2024). Beyond this inhibitory effect on UV-induced inflammation, studies have also found that a sea buckthorn fruit mixture (SFB) can address skin aging issues caused by UV exposure, reducing wrinkles and improving skin texture, firmness, and hydration, while simultaneously downregulating the expression of matrix metallo proteinases (MMPs) and preventing oxidative and photo-damage (Hwang et al., 2012). Collectively, these findings underscore the crucial role of sea buckthorn-derived metabolites in mitigating skin oxidative stress, primarily via the Nrf2 antioxidant pathway.

#### 3.1.2 Mitigating oxidative stress in mucosal tissues

Mucosal tissues are frequently exposed to oxidative challenges from various sources, including pathogens, chemicals, and drugs, which can lead to tissue damage and inflammation (Bhattacharyya et al., 2014). Evidence from studies on sea buckthorn demonstrates its potential to alleviate such oxidative stress. A study using a chronic obstructive pulmonary disease (COPD)-like mouse model established by long-term cigarette smoke exposure combined with intratracheal lipopolysaccharide (LPS) challenge confirmed that total flavonoids from sea buckthorn (TFSB) could alleviate inflammatory responses by activating the Nrf2 pathway and upregulating protective factors in the lungs (Xu et al., 2022). Furthermore, in a mouse model of LPS-induced acute lung injury, Sea buckthorn paste (SP), a traditional Tibetan medicine with high polyphenol content, significantly attenuated oxidative stress and lung tissue injury by promoting Nrf2 nuclear translocation and activation, manifested by suppressing the accumulation of the lipid peroxidation product malondialdehyde (MDA) and enhancing the levels of superoxide dismutase (SOD) and glutathione peroxidase (Du et al., 2017). Similarly, in a monocrotaline (MCT)-induced pulmonary arterial hypertension (PAH) rat model, Isorhamnetin (ISO)—a major natural flavonoid extracted from sea buckthorn—improved hemodynamic parameters and pulmonary vascular remodeling by upregulating Nrf2 protein expression in lung tissue, enhancing SOD activity, and inhibiting the NADPH oxidase 1 (NOX1) and p-c-src signaling pathways, thereby alleviating oxidative stress (Chen Y. et al., 2024).

In the digestive tract, in the context of *Helicobacter pylori* infection, sea buckthorn organic acid extracts (SOA) was shown to reduce ROS and pro-inflammatory cytokines, such as interleukin (IL)-β, IL-6, and IL-8, in gastric mucosal cells, indicating its direct role in countering oxidative and inflammatory pathways (Gao et al., 2025). Similarly, in rat models of methotrexate (MTX)-induced oral and oropharyngeal mucositis, sea buckthorn extract significantly lowered MDA levels—a marker of lipid peroxidation—and increased total glutathione (tGSH), an antioxidant, while suppressing inflammatory cytokines such as IL-1β and tumor necrosis factor -alpha (TNF-α) at both biochemical and gene expression levels (Dilber et al., 2023; Kuduban et al., 2016). Additionally, in an alcohol-induced intestinal barrier dysfunction model, flavonoids from *Hippophae rhamnoides* ssp. *Sinensis* seed residue (HRSF) attenuated oxidative stress (e.g., by inhibiting ROS and MDA accumulation and enhancing glutathione levels and SOD activity) and enhanced the expression of tight junction proteins (e.g., occludin and zona occludens 1 (ZO-1)) by modulating the Nrf2-mediated pathway, thereby protecting intestinal barrier integrity (Wei et al., 2023). Likewise, in high-fat diet (HFD)-fed zebrafish, Sea Buckthorn Polysaccharide (SP) enhanced the antioxidant capacity of the liver and intestine by upregulating Nrf2 expression (e.g., increasing Cu/Zn-SOD activity) and improved gut microbiota balance, thereby reducing lipid accumulation and inflammatory responses (Lan et al., 2022).

### 3.2 Targeting the core inflammatory signaling hub: NF-κB and MAPK pathways

The initiation and amplification of inflammation are governed by key signaling pathways, such as NF-κB and mitogen-activated protein kinases (MAPK) (Ghosh and Hayden, 2008; Huang et al., 2010; He B.-F. et al., 2023). Suppressing these pathways is critical for curbing the excessive release of pro-inflammatory mediators and preventing the amplification of the inflammatory cascade. Sea buckthorn metabolites demonstrate potent inhibitory effects on these hubs, albeit with tissue-specific nuances.

#### 3.2.1 Suppression of pro-inflammatory signaling in skin disorders

In skin inflammation, the NF-κB and MAPK pathways are activated in keratinocytes and immune cells by various triggers, driving pathologies like PSO and AD (Hao et al., 2023; Zhang et al., 2023; Ni et al., 2022; Yang et al., 2021). Sea buckthorn derivatives have been shown to target these pathways effectively. For instance, in a dinitrochlorobenzene (DNCB)-induced AD-like mouse model, topical application of sea buckthorn oil ameliorated dermatitis severity, reduced epidermal thickness, and suppressed mast cell infiltration by inhibiting NF-κB, janus kinase 2/signal transducer and activator of transcription 1 (JAK2/STAT1), and p38-MAPK signaling, thereby reducing the production of T helper cell 2 (Th2) chemokines thymus and activation-regulated chemokine (TARC) and MDC (Hou et al., 2017). Similarly, in a tissue plasminogen activator (TPA)-induced PSO-like model, sea buckthorn oil (SBKT) demonstrated anti-psoriatic efficacy by downregulating NF-κB expression and subsequent pro-inflammatory cytokines such as IL-1β, IL-6, and TNF-α, leading to reduced ear edema and epidermal thickness (Balkrishna et al., 2019). Additionally, casuarinin, a tannin isolated from sea buckthorn, suppressed TNF-α/interferon (IFN)-γ-induced expression of TARC and MDC in human keratinocytes (HaCaT cells) by blocking p38 MAPK activation and subsequent inhibition of NF-κB and STAT1, highlighting its potential as a therapeutic agent for inflammatory skin diseases (Kwon et al., 2012).

#### 3.2.2 Inhibition of signaling pathways in mucosal inflammation

In mucosal tissues, such as the intestine, these pathways are often triggered by microbial components (De Plaen et al., 2006; O'Mahony et al., 2008; Böhringer et al., 2016). Sea buckthorn metabolites exhibit protective effects by modulating these signaling cascades. For example, in LPS-induced porcine intestinal epithelial cells (IPEC-J2), pre-treatment with sea buckthorn polysaccharide (HRP) reduced inflammatory factors (e.g., IL-1β, IL-6, IL-8, TNF-α) and apoptosis while enhancing tight junction proteins and immunoglobulins, via inhibition of the Toll-like receptor 4 (TLR4)/myeloid differentiation factor 88 (MyD88)/NF-κB pathway (Zhao et al., 2020b). Proteomic analysis further confirmed that HRP pre-treatment alleviated LPS-induced inflammation by modulating differentially expressed proteins involved in immune-related pathways such as MAPK and NF-κB, with Western blotting validating the reduction in phosphorylated MAPK7, reticuloendotheliosis viral oncogene homolog A (RELA), NF-κB1, and NF-κB2 (Zhao et al., 2020a). In respiratory mucosa, TFSB prevented LPS/cigarette smoke-induced airway inflammation in mice and HBE16 bronchial epithelial cells by suppressing the expression of IL-1β, IL-6, chemokine (C-X-C motif) ligand 1 (CXCL1), and mucin 5 subtype AC (MUC5AC) through inhibition of extracellular regulated protein kinases (ERK), phosphoinositide 3-kinase/protein kinase B (PI3K/Akt), and protein kinase C-alpha (PKCα) pathways, as supported by molecular docking studies showing binding affinities to upstream kinases (Ren et al., 2019).

### 3.3 Modulating immune cell responses and cytokine networks

Following initial signaling, inflammation is characterized by the recruitment and activation of specific immune cells and the release of cytokines (Panigrahy et al., 2021). Regulating the metabolism and breakdown of these pro-inflammatory mediators is crucial to prevent persistent inflammation and facilitate resolution (Lawrence et al., 2002). Sea buckthorn modulates this intermediate layer of the immune response to restore balance.

#### 3.3.1 Balancing immune responses in cutaneous inflammation

Skin inflammation involves dysregulated crosstalk between various immune cells (Zouboulis et al., 2022; Honda and Kabashima, 2020). Sea buckthorn and its bioactive components have demonstrated efficacy in rebalancing immune responses in models of AD and PSO. For instance, in a DNCB-induced AD-like mouse model, topical application of sea buckthorn oil ameliorated dermatitis severity, reduced epidermal thickness, decreased spleen and lymph node weights, and suppressed mast cell infiltration, primarily through dose-dependent inhibition of Th2 chemokines TARC and MDC via NF-κB, JAK2/STAT1, and p38-MAPK signaling pathways (Hou et al., 2017). Similarly, in a PSO-like model induced by TPA in mice, sea buckthorn oil exhibited anti-inflammatory and anti-psoriatic effects by reducing ear edema and skin lesion scores, suppressing NF-κB activation, and inhibiting pro-inflammatory cytokines such as TNF-α, IL-1β, and IL-6 (Balkrishna et al., 2019). Another key component, isorhamnetin (IRh), derived from sea buckthorn, alleviated imiquimod (IMQ)-induced psoriasiform lesions in mice by reducing oxidative stress (e.g., lowering MDA and restoring SOD and catalase (CAT) levels), suppressing pro-inflammatory cytokines (TNF-α, IL-6, IL-17A), and modulating T helper cell 1 (Th1) and T helper cell 17 (Th17) cell populations and dendritic cell maturation (Wu et al., 2022). Furthermore, topical application of total flavonoids from sea buckthorn (TFH) in an Calcipotriol (MC903)-induced AD model improved skin barrier function by upregulating filaggrin expression, reduced mast cell infiltration, and balanced Th1/Th2 cytokines (e.g., decreasing TNF-α, IL-4, IFN-γ, and thymic stromal lymphopoietin (TSLP)), while inhibiting NF-κB, ERK, and p38 signaling in keratinocytes (Gu et al., 2022). Additionally, extracts from sea buckthorn fruit peel showed anti-allergic effects in metabolite 48/80-induced paw edema by stabilizing mast cell membranes and inhibiting degranulation, with ursolic acid and oleanolic acid identified as active metabolites (Rédei et al., 2018). These studies collectively underscore sea buckthorn’s ability to modulate cutaneous immune responses through multiple pathways, including chemokine suppression, oxidative stress reduction, and cytokine network rebalancing.

#### 3.3.2 Regulating mucosal immunity and microbiota crosstalk

Mucosal immunity is distinguished by its intimate interaction with the microbiota (Mowat and Agace, 2014; Shi et al., 2017). Sea buckthorn exerts regulatory effects on mucosal immunity by influencing gut microbiota composition, barrier function, and immune cell activity. In an intestinal model using colorectal adenocarcinoma (Caco-2)cells, an aqueous extract of sea buckthorn fruits inhibited LPS leakage through the epithelial monolayer, reduced pro-inflammatory cytokine secretion (e.g., IL-8, IL-1β, IL-6, TNF-α), and maintained glucose transporter protein 2 (GLUT2) expression, suggesting a role in preventing metabolic endotoxemia and low-grade inflammation (Laskowska et al., 2022). In a COPD rat model induced by LPS and smoking, Sea buckthorn Wuwei Pulvis (SWP)—a traditional Mongolian medicine containing sea buckthorn—improved pulmonary function (e.g., Forced Expiratory Volume (FEV)0.3/Forced Vital Capacity (FVC) ratio), reduced lung inflammatory cytokines (TNF-α, IL-8, IL-6, IL-17), and enhanced intestinal barrier integrity by upregulating tight junction proteins (ZO-1 and occludin-1). These effects were linked to modulation of gut microbiota (e.g., increased Ruminococcaceae and Christensenellaceae) and elevated short-chain fatty acid (SCFA) production (Wang et al., 2023). Moreover, in immunosuppressed mice induced by cyclophosphamide, sea buckthorn pulp oil enhanced systemic and mucosal immunity by promoting T lymphocyte proliferation, NK cell cytotoxicity, macrophage phagocytosis, and cytokine production (e.g., IFN-γ, IL-2, IL-4, IL-12, TNF-α, and secretory Immunoglobulin A (sIgA)). This was accompanied by increased SCFA levels and restoration of gut microbiota diversity, with elevated beneficial genera (e.g., *Lactobacillus* and Roseburia) and reduced pathobionts (e.g., Mucispirillum and *Acinetobacter*) (Zhang et al., 2021). In a zebrafish model of foodborne enteritis, sea buckthorn supplementation alleviated intestinal and hepatic inflammation by improving villi morphology, reducing immune cell infiltration (e.g., neutrophils and T cells), and modulating gut microbiota (e.g., reducing TM7 and *Shigella*). It also inhibited the p53 signaling pathway in the intestine while activating peroxisome proliferator activated receptor (PPAR) signaling and fatty acid metabolism in the liver, highlighting its role in gut-liver axis regulation (Li M. et al., 2022). These findings demonstrate that sea buckthorn stabilizes mucosal immunity by reinforcing barrier function, shaping microbial communities, and fine-tuning immune cell responses through SCFA and cytokine networks.

### 3.4 Promoting tissue repair and barrier restoration

The resolution of inflammation culminates in tissue repair and remodeling (Wang Z.-C. et al., 2020; Kaplani et al., 2018). This phase is critical for restoring barrier integrity and function, representing the final step in resolving the inflammatory process (Livshits and Kalinkovich, 2024). Sea buckthorn promotes healing through effects on various cell types and structural components, with processes differing between skin and mucosa.

#### 3.4.1 Enhancing healing and angiogenesis in skin wounds

Skin repair involves re-epithelialization, collagen deposition, and angiogenesis (Sarate et al., 2024). Sea buckthorn extracts and oils have been extensively studied for their efficacy in enhancing these processes. For instance, seed oil enriched with saturated and unsaturated fatty acids, such as palmitic acid, promotes the proliferation of normal keratinocytes and skin fibroblasts, indicating regenerative properties on skin cells (Dudau et al., 2021). Topical application of sea buckthorn flavone accelerates wound contraction and epithelialization in rat models, as evidenced by reduced epithelialization time (16.3 days vs. 24.8 days in controls) (Gupta et al., 2006). Preclinical studies on leaf extracts further support wound healing, with polyvinyl alcohol-blended pectin hydrogel containing leaf extracts significantly reducing wound size and improving recovery rates in rats (Kim and Lee, 2017). In burn wound models, lyophilized aqueous leaf extract enhances collagen synthesis and stabilization, upregulates collagen type-III expression, and promotes angiogenesis via increased vascular endothelial growth factor (VEGF) expression, while also boosting antioxidant levels and reducing lipid peroxidation (Upadhyay N. K. et al., 2009). Similarly, supercritical CO_2_-extracted seed oil augments burn wound healing by increasing wound contraction, hydroxyproline, hexosamine, DNA, and total protein content, up-regulating matrix metalloproteinases (MMP-2 and -9), collagen type-III (Upadhyay N. et al., 2009). In large animal models, seed oil application to full-thickness burns and donor sites in sheep significantly improves epithelization rates (95% ± 2.2% vs. 83% ± 2.9%) and shortens complete epithelization time (14.20 ± 0.48 days vs. 19.60 ± 0.40 days), confirming its efficacy in promoting re-epithelialization and graft healing (Ito et al., 2014). These findings collectively demonstrate that sea buckthorn components, including fatty acids, flavones, and leaf extracts, enhance skin repair through multiple mechanisms, including cell proliferation, collagen deposition, antioxidant activity, and angiogenesis.

#### 3.4.2 Accelerating repair and mucosal integrity restoration

Mucosal repair focuses on rapid epithelial renewal and restoration of barrier function (Chen et al., 2022; Kuo et al., 2021). Sea buckthorn has shown promise in accelerating mucosal healing, particularly in gastric ulcer models. CO2-extracted seed and pulp oils, when administered orally, significantly reduce ulcer formation in water-immersion and reserpine-induced models in rats, and accelerate the healing of acetic acid-induced gastric ulcers, indicating both preventive and curative effects (Xing et al., 2002). Further, sea buckthorn procyanidins (SBPC) from bark extract enhance the healing of acetic acid-induced gastric lesions by reducing ulcer size in a dose-dependent manner, increasing epidermal growth factor (EGF) levels in plasma, and up-regulating the expression of epidermal growth factor receptor (EGFR) and proliferating cell nuclear antigen (PCNA) around the ulcer site, which promotes mucosal repair and epithelial renewal (Xu et al., 2007). These studies highlight the role of sea buckthorn oils and procyanidins in restoring mucosal integrity through enhanced epithelial cell proliferation and growth factor signaling. Table 1 presents the experimental models and mechanisms of skin and mucosal inflammation modulation by sea buckthorn and its derivatives.

**TABLE 1:** Experimental models and mechanisms of skin and mucosal inflammation modulation by sea buckthorn and its derivatives.

Study types			Sea buckthorn and its derivatives	Disease types	*In vitro*/*in vivo*/clinical	Models	Cellular targets	Mechanism	References
experimental research	preclinical research	*in vitro*	Sea buckthorn oil	PSO	*in vitro*	human monocytic cells	NF-κB	downregulation of NF-κB protein and pro-inflammatory cytokines	Balkrishna et al. (2019)
			Sea buckthorn seed Oil	UV damage	*in vitro*	human keratinocytes and fibroblasts	Nrf2、PPAR and cannabinoid receptor	disturbances in redox balance and lipid metabolism	Gęgotek et al. (2018)
			Sea buckthorn polysaccharide	intestinal structural abnormalities and dysfunction	*in vitro*	IPEC-J2	IPEC-J2	inhibiting TLR4/NF-κB signaling pathway	Zhao et al. (2020b)
			Fatty acid	(skin cell regeneration)	*in vitro*	normal skin cells	IL-8,VEGF	supported cell proliferation for keratinocytes and dermal fibroblasts	Dudau et al. (2021)
		*in vivo*	Sea buckthorn	gut-liver Immune axis	*in vivo*	zebrafish	not mentioned	enhancing intestinal mucosal immunity and microbiota while inhibiting hepatic adipose disposition	Li Y.-Y. et al. (2022)
			Sea buckthorn oil	AD-like skin lesions	*in vivo*	mice	NF-κB and STAT1	inhibition of the Th2 chemokines TARC and MDC	Hou et al. (2017)
			Sea buckthorn seed Oil	burn wounds	*in vivo*	adult sheep	not mentioned	promote skin and mucosa epithelization	Ito et al. (2014)
			Sea buckthorn pulp oil	immunosuppression	*in vivo*	mice	sIgA, IFN-γ, IL-2, IL-4, IL-12 and TNF-α	regulate the diversity and composition of intestinal microflora	Zhang et al. (2021)
				gastric ulcer	*in vivo*	rats	not mentioned	undefined	Xing et al. (2002)
			Sea buckthorn extracts	5-FU-associated oral mucositis	*in vivo*	rats	NF-κB, IL-6	antagonizing the effects of 5-FU on oxidant, antioxidant and proinflammatory cytokines	Dilber et al. (2023)
				oral mucositis	*in vivo*	rats	IL-1β, TNF-α	prevent the increase of MDA and decrease of tGSH	Kuduban et al. (2016)
			Sea buckthorn leaf extracts	burn wounds	*in vivo*	rats	COL-3,VEGF,MMP-2 and -9	augmented endogenous antioxidants and prevented the free-radical-mediated tissue injury	Upadhyay N. K.et al. (2009)
				wound	*in vivo*	albino rats	hydroxyproline, hexosamine	increased antioxidant levels	Gupta et al. (2005)
			Sea buckthorn fruit extracts	UV radiation-induced skin aging	*in vivo*	hairless mice	collagen-1,MMP-1 and -9	regulating the moisture content, MMP expression levels and SOD activity	Hwang et al. (2012)
			Sea buckthorn polysaccharide	intestinal disorders and dysfunction	*in vivo*	IPEC-J2	IPEC-J2	inhibiting the MAPK/NF-κB signaling pathway	Zhao et al. (2020a)
			Isorhamnetin	COPD	*in vivo*	mice	IL-6,RANTES and MCP-1	affecting the Nrf2/Keap1 pathway	Xu et al. (2022)
				Psoriasiform Dermatitis	*in vivo*	mice	TNF-α, IL-6, IL-17A, and NF-κB	decreasing TNF-α, IL-6, IL-17A, and nuclear NF-κB levels in the skin	Wu et al. (2022)
			Sea buckthorn flavonoids	AD-like lesions	*in vivo*	mice	FLG,TSLP,IFN-γ,IL-4,TNF-α,p-NF-κB, p-ERK and p-P38	regulating Th1/Th2 balance	Gu et al. (2022)
				dermal wound	*in vivo*	rats	hydroxyproline, hexosamine	improved wound contraction and epithelialization, increase hydroxyproline and hexosamine content	Gupta et al. (2006)
			Ursolic acid and oleanolic acid	edema	*in vivo*	rats	mast cells	inhibition of degranulation of mast cells	Rédei et al. (2018)
			procyanidins	Chronic gastric ulceration	*in vivo*	rats	EGF, EGFR, PCNA	accelerated mucosal repair	Xu et al. (2007)
			Poly-herbal formulation	diabetic wound	*in vivo*	rats	VEGF, MMP-2,hydroxyproline, hexosamine	increase angiogenesis; increase proliferation and transformation of fibroblast cells into myofibroblasts	Gupta et al. (2006)
			Sea buckthorn Wuwei Pulvis	COPD	*in vivo*	mice	TNF-α,IL-8,IL-6,IL-17,ZO-1 and occludin-1	shaping the gut microbiota and increasing SCFA production	Wang et al. (2023)
		*in vivo/in vitro*	Sea buckthorn seed Oil	burn wounds	*in vivo/in vitro*	rats	MMP-2 and 9, collagen type-III and VEGF	increase in wound contraction, hydroxyproline, hexosamine, DNA and total protein contents	Upadhyay N. et al. (2009)
	clinical research	Randomized controlled trail	Sea buckthorn	second-degree burns	clinical	human	not mentioned	undefined	
			Sea buckthorn seed and pulp oils	AD	clinical	human	not mentioned	increased the proportion of docosapentaenoic acid and decreased the proportion of palmitic acid	Yang et al. (2000)
			Sea buckthorn fruit extracts	acne	clinical	healthy human	not mentioned	inhibiting type 1-α reductase to regulate sebum secretion	Akhtar et al. (2010)

## 4 Further perspectives

This review underscores the significant anti-inflammatory properties of sea buckthorn and its derivatives, which operate through multiple primary mechanisms: the inhibition of core signaling pathways, modulation of immune responses, activation of endogenous antioxidant systems, and promotion of tissue repair. Our analysis demonstrates that sea buckthorn compounds interact with multiple biological targets, including TNF-α, NF-κB, MAPK, MMP-2, and Nrf2. These interactions suggest that sea buckthorn may play a crucial role in managing inflammatory conditions such as AD, PSO, burns, and UV-induced damage.

Despite promising preclinical findings, the clinical translation of sea buckthorn requires systematic investigation. Compelling evidence from animal studies has elucidated the mechanisms of its key bioactive compounds. For instance, sea buckthorn flavonoids and unsaponifiables have demonstrated significant efficacy in ameliorating symptoms of AD in murine models, including reduction of erythema, immune cell infiltration, and barrier damage, primarily through modulation of NF-κB and MAPK signaling pathways. However, translating these findings into clinical applications faces considerable challenges.

Firstly, the complex composition of sea buckthorn presents a major obstacle to its standardization. The content and ratio of its active components (such as flavonoids, fatty acids, vitamins) vary significantly depending on the plant part (e.g., fruit, seed, leaf) and geographical origin. This inherent variability poses serious challenges for product quality control and batch-to-batch consistency. Therefore, establishing robust chemical fingerprints based on chemical pattern recognition technology and implementing stringent quality standards for different medicinal parts (e.g., fruit extracts, seed oil) are crucial for ensuring the efficacy and safety of sea buckthorn products.

Secondly, regarding its mechanism of action, a fundamental question remains unresolved: whether the observed pharmacological effects are attributable to single components or result from synergistic interactions among multiple metabolites. Current research predominantly focuses on crude extracts or mixtures (e.g., sea buckthorn oil), making it difficult to pinpoint the specific contributors. Future research needs to focus on isolating and identifying single active metabolites (e.g., isorhamnetin, specific flavonoid glycosides) and rigorously validating their independent effects and potential synergies through pharmacodynamic and mechanistic studies in both animal and clinical models.

Furthermore, the poor bioavailability and formulation instability of some key compounds are significant hurdles on the path to translation. This directly impacts their *in vivo* absorption and ultimate efficacy. To address this, adopting advanced drug delivery strategies is imperative. For example, encapsulating active metabolites using nano-carrier systems (such as nanoemulsions, liposomes, polymeric nanoparticles) can improve their water solubility, enhance skin or mucosal permeability, increase stability, and achieve controlled release, thereby significantly enhancing bioavailability and therapeutic effect. Additionally, other dosage form optimizations, such as developing microneedle patches for transdermal delivery or designing mucoadhesive formulations for oral or gastrointestinal lesions, are worthwhile directions to explore.

Finally, the current gap in clinical evidence limits its widespread clinical acceptance and application. As summarized in Table 1, although small-scale clinical trials for conditions like AD and burn healing have reported positive outcomes, these studies are often constrained by limitations such as small sample sizes, lack of rigorous blinding, and short follow-up periods, preventing definitive conclusions regarding efficacy and safety. Consequently, there is an urgent need for large-scale, multi-center, randomized, double-blind, placebo-controlled clinical trials to robustly validate the efficacy and long-term safety of sea buckthorn preparations (particularly highly standardized sea buckthorn oils) in well-defined patient populations (e.g., patients with moderate AD).

**FIGURE 3:**
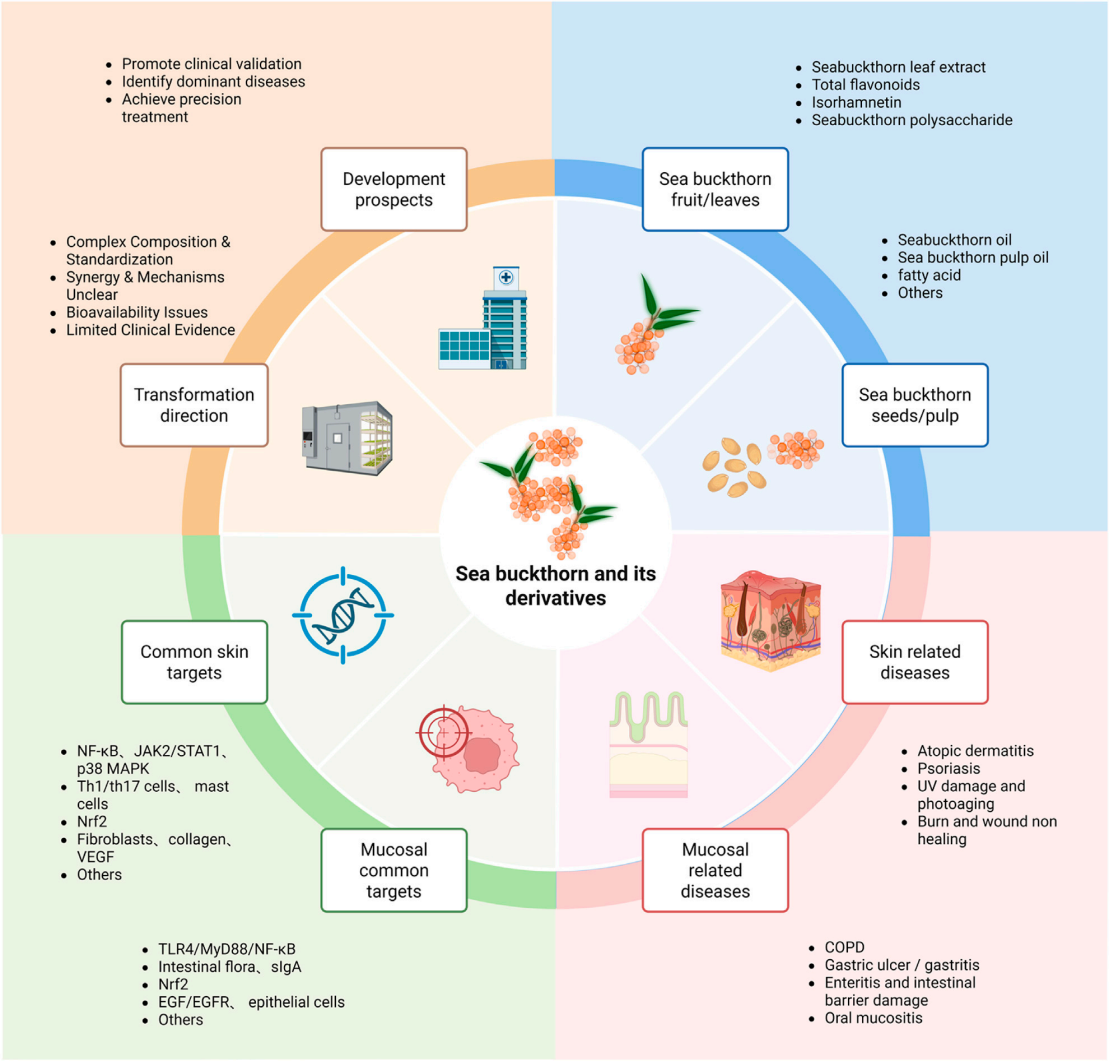
The blue section illustrates the foundation, including bioactive components derived from different plant parts. The pink section demonstrates current therapeutic applications for various diseases. The green section summarizes common molecular targets of sea buckthorn in both skin and mucosal diseases. The yellow section outlines a two-stage future pathway: the Translation phase focuses on overcoming key research bottlenecks (standardization, mechanism elucidation, formulation), while the Development phase details subsequent actions for clinical application (rigorous trials, precision indication, personalized therapy).

Future research should prioritize several key directions. First, conducting more of the high-quality clinical trials mentioned above to verify the efficacy and long-term safety of sea buckthorn oil in patients with moderate AD. Second, studies must explore the optimal administration route and dosage for sea buckthorn active components in the prevention and treatment of other inflammatory conditions, such as oral mucositis. Finally, employing advanced drug delivery strategies (including nanotechnology to enhance bioavailability) and developing personalized treatment approaches based on individual patient profiles and specific inflammatory disorders represent critical frontiers for innovation (see Figure 3).

Bridging the gap from compelling laboratory research to definitive clinical practice holds significant promise. A focused effort on elucidating mechanisms, optimizing formulations, and conducting rigorous clinical trials will pave the way for innovative, nature-derived strategies to improve outcomes for patients suffering from inflammatory skin and mucosal diseases.

## 5 Conclusion

In conclusion, sea buckthorn represents a rich source of multi-target anti-inflammatory agents. This review has systematically organized its mechanisms within the framework of the inflammatory cascade, highlighting its potential from stimulus removal to tissue remodeling. Future research should adopt a targeted approach that prioritizes clinical translation. By integrating advanced pharmaceutical technologies with a deeper understanding of sea buckthorn’s active metabolites, researchers can develop more effective and personalized therapeutic strategies for barrier-related inflammatory diseases.

## Glossary

AD: atopic dermatitis
Caco-2: colorectal adenocarcinoma
CAT: catalase
COL-3: collagen type III
COPD: chronic obstructive pulmonary disease
CXCL1: chemokine (C-X-C motif) ligand 1
DNCB: dinitrochlorobenzene
EGF: epidermal growth factor
EGFR: epidermal growth factor receptor
ERK: extracellular regulated protein kinases
FEV: Forced Expiratory Volume
FVC: Forced Vital Capacity
GLUT2: glucose transporter protein 2
HaCaT: human immortal keratinocyte line
IFN: interferon
IL: interleukin
IMQ: imiquimod
IPEC-J2: porcine intestinal epithelial cells
IRh: isorhamnetin
JAK2/STAT1: janus kinase 2/signal transducer and activator of transcription 1
LPS: lipopolysaccharide
MAPK: mitogen-activated protein kinases
MC903: Calcipotriol
MDA: malondialdehyde
MMP: matrix metallo proteinase
MMPs: matrix metallo proteinases
MTX: methotrexate
MUC5AC: mucin 5 subtype AC
MyD88: myeloid differentiation factor 88
NF-κB: nuclear factor-kappa B
NOX1: NADPH oxidase 1
Nrf2: nuclear red-family 2-related factor
PCNA: proliferating cell nuclear antigen
PI3K/Akt: phosphoinositide 3-kinase/protein kinase B
PKCα: protein kinase C-alpha
PPAR: peroxisome proliferator activated receptor
PSO: psoriasis
RELA: reticuloendotheliosis viral oncogene homolog A
ROS: reactive oxygen species
SBKT: neutral sea buckthorn oil
SCFA: short-chain fatty acids
SFB: sea buckthorn fruit blend
sIgA: secretory Immunoglobulin A
SOA: sea buckthorn organic acid extracts
SWP: Sea buckthorn Wuwei Pulvis
TARC: thymus and activation-regulated chemokine
TFSB: total flavonoids of sea buckthorn
TSLP: thymic stromal lymphopoietin
tGSH: total glutathione
Th1: T helper cell 1
Th2: T helper cell 2
Th17: T helper cell 17
TLR4: Toll-like receptor 4
TNF -α: tumor necrosis factor -alpha
TPA: tissue plasminogen activator
VEGF: vascular endothelial growth factor
ZO-1: zona occludens 1
